# Cyclooxygenase-2 overexpression abrogates the antiproliferative effects of TGF-*β*

**DOI:** 10.1038/sj.bjc.6604048

**Published:** 2007-10-23

**Authors:** G A Enders

**Affiliations:** 1 Institute for Surgical Research, LMU-München, Marchioninistr. 27, München D-81366, Germany

**Keywords:** cyclooxygenase-2, TGF-*β* signal, Smad-pathway, TGF-*α*, PPAR

## Abstract

The influence of cyclooxygenase-2 (COX-2) overexpression on the development of tumours has been well documented. The underlying mechanism however has still not been completely elucidated. An escape of proliferating cells from the regulatory influence of TGF-*β* for example in the intestine has been discussed as well as a preponderance or prolongation of growth factor stimulation. The experiments presented here demonstrated that COX-2 transfection of a TGF-*β*-sensitive cell line abrogates the growth inhibitory effects of TGF-*β*. However, analysis of the TGF-*β*/Smad-signalling pathway clearly revealed that COX-2 overexpression did not interfere with that. Neither TGF-receptor expression nor Smad phosphorylation and signal transfer into the nucleus were influenced by COX-2 overexpression. In addition, a TGF-*β* reporter assay revealed no difference between controls and COX-2-transfected cells. Thus, the proliferation inhibiting effects must have been well compensated by growth-inducing stimuli. Indications for this came from experiments showing an induction of TGF-*α* expression and secretion with a higher and prolonged stimulation of the ERK 1/2 (p42/44) pathway in COX-2 transfectants. This effect could have been triggered by direct prostaglandin receptor stimulation or changes in intracellular lipid mediators. An increase in PPAR signalling as proven by a reporter assay is indication for the latter. Therefore, inhibiting both COX-2 as well as the PPAR and TGF/EGF pathway could be effective in the inhibition of adenoma or even carcinoma development in the intestine.

The inducible cyclooxygenase-2 (COX-2) has been shown to be overexpressed in colorectal cancer and adenomas in humans and was demonstrated to be a controlling factor in the development of adenomas in a mouse FAP model (Eberhart *et al*, 1994; Oshima *et al*, 1996). In addition, the regular use of COX inhibitors has been shown to reduce the relative risk for the development of colon cancer (Thun *et al*, 1991; Greenberg *et al*, 1993; Steinbach *et al*, 2000; Baron *et al*, 2003; Sandler *et al*, 2003). The mechanistic steps, which link COX-2 overexpression with the generation of a tumour are however still not fully understood. Possible pathways involve the escape from regulatory mechanisms as well as interference with stimulatory effects on cell proliferation. The former comes from experiments in rat cells showing a reduction of the TGF-*β*-receptor expression after COX-2 overexpression (Tsujii and DuBois, 1995). As TGF-*β* has a major function as regulator for the cell renewal in the intestine, this notion gains some support. In addition, mutations in the TGF-*β*-receptor have also been associated with certain tumours in the intestine (Parsons *et al*, 1995). On the other hand, mutations are not as common as one would expect, so regulatory deficits should be much more relevant. To this end, no functional consequences of COX-2 overexpression in the TGF-*β*-pathway have been shown with regard to growth inhibition or signal transduction. Therefore, we tested the effects of COX-2 overexpression on the growth characteristics of a highly TGF-*β*-responsive cell line. In addition, we analysed the TGF-*β* signalling pathway and also the influence of COX-2 overexpression on growth stimulatory mediators.

## MATERIALS AND METHODS

### Cell culture

The Mv1Lu mink lung epithelial cell line and the human CRC line HCT-15 were from ATCC (Manassas, VA, USA). Cells were grown in RPMI 1640 supplemented with 1% glutamine, 1% non-essential amino acids, 1% MEM vitamins and 10% FCS (all from Invitrogen, Karlsruhe, Germany).

### Transfection

Full-length COX-2 from IL-1-induced human endothelial cells as well as cPLA_2_ from LPS stimulated U937 (human monocytic cell-line) were amplified and cloned into the pcDNA3 expression vector (Invitrogen, Karlsruhe, Germany). After sequence validation, plasmid DNA was transfected using FuGene-transfection reagent according to the protocol of the manufacturer (Roche, Mannheim, Germany).

### TGF-stimulation

Control cells (10^5^) as well as COX-2-transfected cells were stimulated with TGF-*β* (Strathman Biotech, Hannover, Germany) over 24 h. Bromodeoxyuridine (BrdU) was added during the last 4 h and cell proliferation measured by a BrdU-assay (Roche, Mannheim, Germany).

### Western blotting

Proteins were separated on a standard 10% polyacrylamide gel, transferred to nitrocellulose and analysed by incubation with the specific Ab. Ab used were anti-COX-2 (BD Biosciences, Heidelberg and Santa Cruz Biotechnology, Santa Cruz, CA, USA), anti-c-myc (Santa Cruz) and anti-(phospho)-p44/42 (ERK1/2; Cell Signalling Technology, Frankfurt, Germany). For the detection of Smad and phosphorylated Smad, a polyclonal Ab was used (a generous gift from P ten Dijke) (Piek *et al*, 1999). Secondary HRP-coupled Ab as well as the chemiluminescent substrate (picoSignal) was from Pierce/Perbio, Bonn.

### Reporter assay

The TGF-*β* reporter construct 3TP-Lux (Wrana *et al*, 1992) was transiently transfected using FuGene (Roche) into Mv1Lu as well as stable COX-2-transfected cells. Twenty-four hours later, cells were stimulated with TGF-*β* (1 and 10 ng ml^−1^) and lysed after an additional 24 h. Equal amounts of protein were tested for luciferase and normalised for *β*-Gal activity (Microlumat, Berthold, Bad Wildbad; Luciferase/*β*-Gal reporter assay/Roche). As reporter for lipid mediators, the DR1-luciferase reporter pBLTK 1 was used (kindly provided by BU Bauer/Uni Marburg).

### RT–PCR

RNA was isolated 24 h after COX-2 transfection of cells using the RNA easy kit according to the manufacturer's protocol (Quiagen, Hilden, Germany). The cDNA synthesis and PCR was performed by standard methods with primers for human TGF-*α*, HGF and EGF. The correct size gene-products were densitometrically analysed after separation on a 1% agarosegel (1D-Software, LTF, Wasserburg, Germany).

### ELISA

TGF-*α* (R&D Systems, Wiesbaden, Germany) and prostaglandin E_2_ (PGE_2_) (correlate EIA, Assay Designs, Ann Arbor, USA) were measured in the supernatant of cells by commercial ELISAs.

## RESULTS

**Figure 1:**
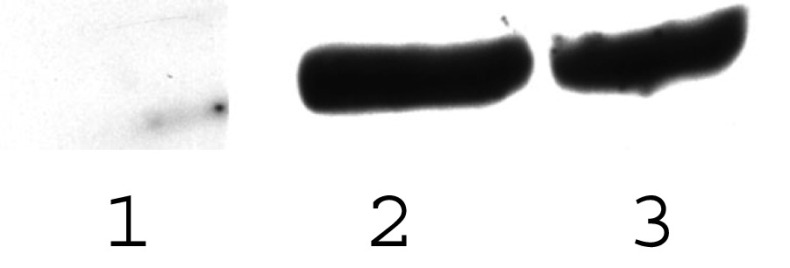
Detection of COX-2 protein by Western blotting. In lane 1, the parental cell line Mv1Lu was analysed and lanes 2 and 3 are 2 separate lines transfected with COX-2.

After stabile transfection of Mv1Lu (CCL-COX), the appearance of the cells changed from an adherent monolayer to a suspension culture. There was no obvious change in viability or doubling time. This morphology was stable over several cell passages. Stabile transfectants express COX-2 as shown by RT–PCR and Western blotting (Figure 1). COX-2 was functional as shown by an increased PGE production from 64.9 to 157.7 pg ml^−1^ (mean from one transient/one stabile TF; measured in triplicate) in controls and transfectants, respectively.

**Figure 2:**
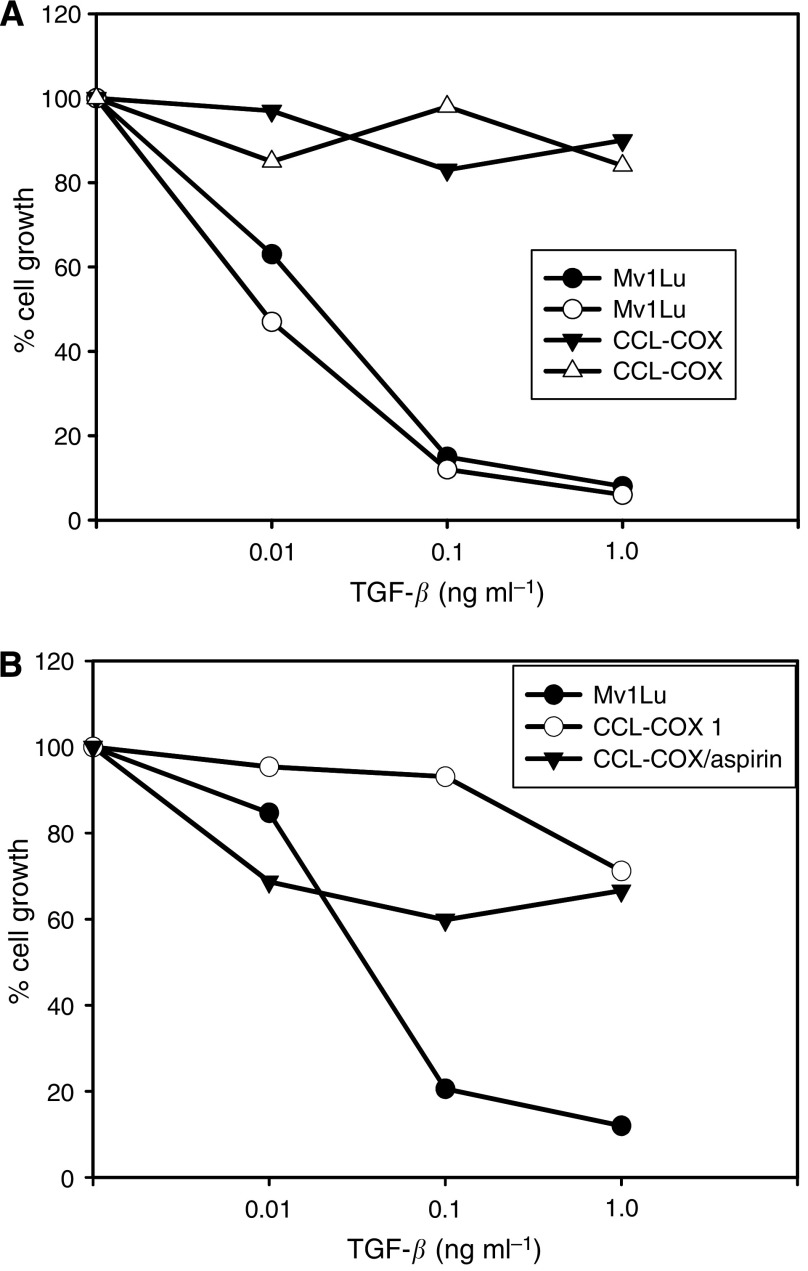
Growth inhibition curve of Mv1Lu and the COX-2 transfected line CCL-COX after TGF-*β* stimulation in (**A**). Proliferation was tested by BrdU incorporation. In (**B**), the effect of aspirin on the growth of TGF-*β*-stimulated COX-2-transfected cell lines is plotted in percent of control growth. Each point was measured in triplicate and the experiment was repeated at least four times.

To test the impact of COX-2 expression on the TGF-*β*-signalling pathway, parental and transfected cell lines were stimulated with TGF-*β*. In accordance with reports from the literature, TGF-*β* inhibited the proliferation of Mv1Lu cells in a concentration-dependent manner. In contrast, COX-2-transfected Mv1Lu cells were refractory to the inhibitory effects of TGF-*β* up to a concentration of 1 ng, which totally blocks proliferation in the original Mv1Lu (Figure 2A). Blocking the cyclooxygenase activity by aspirin could partly reverse this effect (Figure 2B).

**Figure 3:**
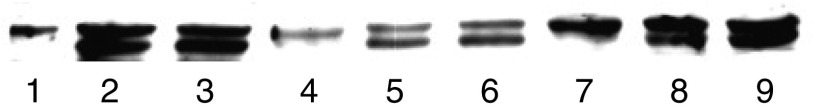
Phosphorylation of Smad after TGF-*β* stimulation of Mv1Lu (lane 1–3) and two different COX-2-transfected cell lines (4–6 and 7–9). Lanes 1, 4 and 7 are cells before TGF-*β* stimulation, 2, 5 and 8 are 15 min and 3, 6 and 9 are 30 min after TGF-*β* (10 ng ml^−1^) stimulation. The lower band after stimulation corresponds to the phosphorylated Smad (lanes 2, 3, 5, 6, 8 and 9).

**Figure 4:**
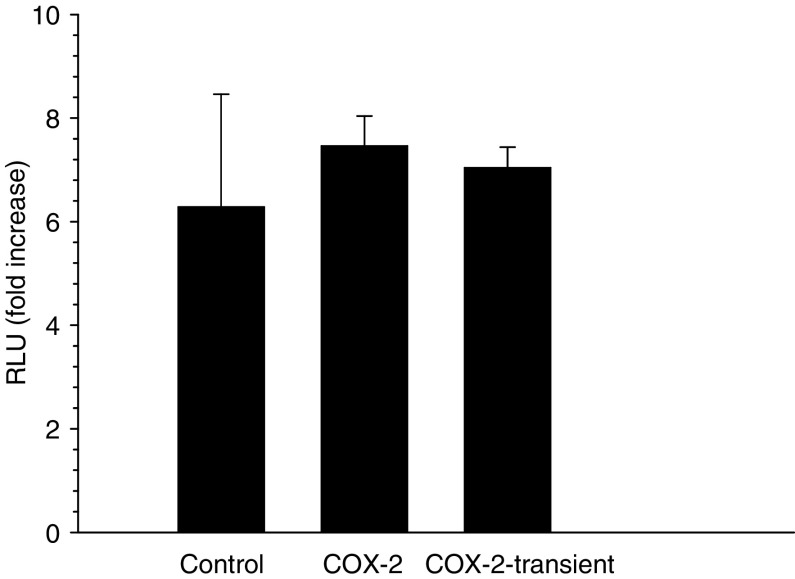
Reporter assay using the TGF-*β*-responsive 3TP-Luc reporter construct. The mean fold increase over control (measured in triplicate) is given of cell lysates after normalisation for *β*-Gal activity. The experiment was repeated once.

The effects of TGF-*β* after binding to its receptors are transmitted via the Smad pathway, where phosphorylated Smad 2/3 enters the nucleus for activation of downstream genes (Heldin *et al*, 1997). Protein analysis by Western blotting did not show any difference in TGF-*β* type I and II receptor expression between control and COX-2 expressing cells (Supplementary Figure 1), which was in contrast to experimental data in a rat epithelial cell line (Tsujii and DuBois, 1995). Furthermore, the Smad-signalling pathway was intact. There was no difference in the expression of Smad 2 and 3 (Supplementary Figure 2) and it was equally phosphorylated after TGF-*β* stimulation in controls and COX-2-transfected cells (Figure 3). In addition, the transfer of the Smad signal into the nucleus after TGF-*β* stimulation could be equally demonstrated by immunofluorescence in control as well as COX-2-transfected cells (Supplementary Figure 3). The functional effect could also be shown by an established TGF-*β* reporter assay (Wrana *et al*, 1992). After co-transfection with the reporter plasmid, the luciferase activity increased in controls as well as in COX-2-transfected cells by about eight times. Thus, this reporter experiment further supports the notion that the TGF-*β* signal via Smad is transmitted in the COX-2 transfectants to the same extend as in controls (Figure 4).

**Figure 5:**
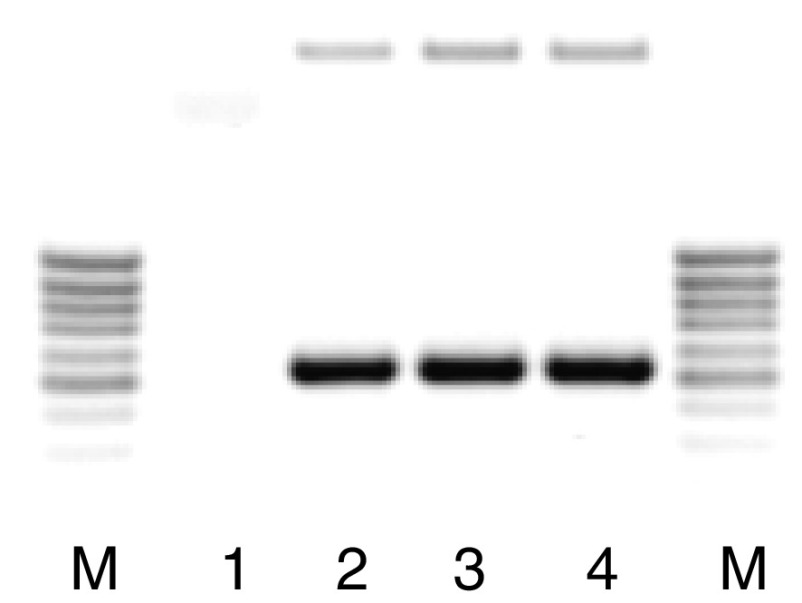
RT–PCR for TGF-*α* of cDNA from control (lane 1), COX-2 (lane 2), cPLA (lane 3) and combined COX-2/cPLA (lane 4)-transfected cells. Amplification was for 30 or 35 cycles and analysed on a 1% agarose gel stained with ethidium bromide. M, MW marker lane; predicted amplicon size is 519 bp.

**Figure 6:**
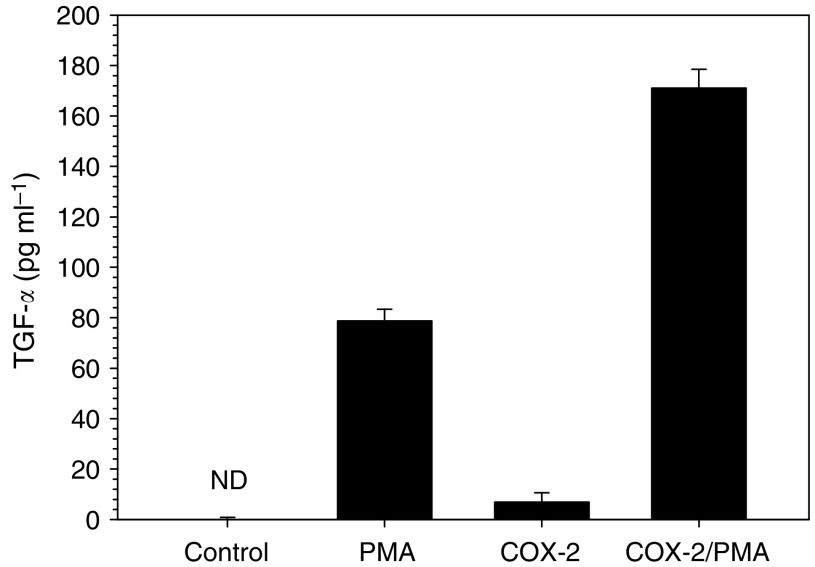
TGF-*α* concentration (pg ml^−1^) in the supernatant of control and COX-2-transfected HCT-15 with and without phorbolester (PMA, 1 ng ml^−1^) stimulation. Concentration was measured in triplicate. Results (mean) are from two independent experiments.

**Figure 7:**
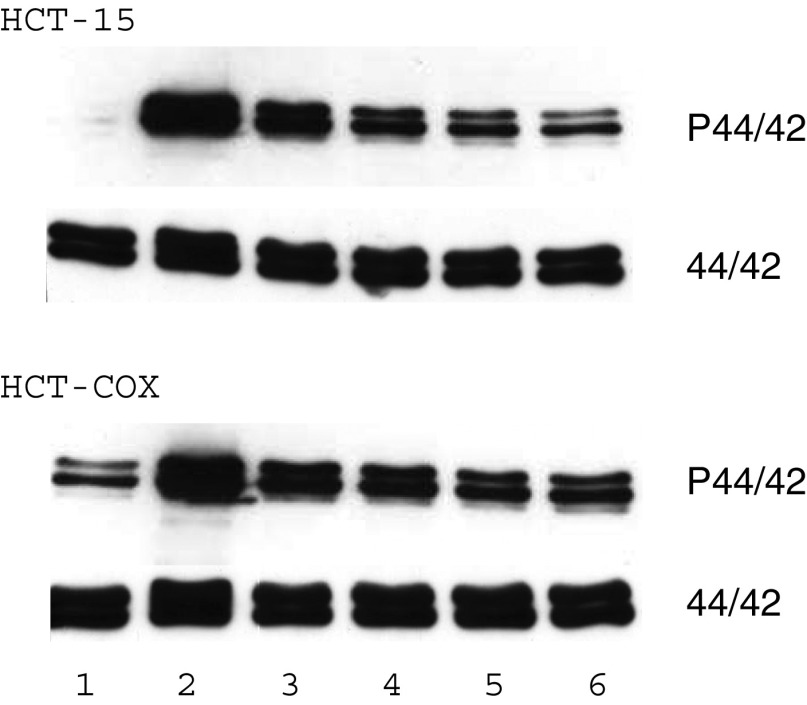
Phosphorylation of ERK1/2 (p42/44) after EGF stimulation in control (first two rows) and COX-2-transfected HCT-15 (third and fourth row). First lane is the control without stimulation, next lanes are 5, 10, 15, 30 and 45 min after stimulation, respectively. In rows 2 and 4, the unphosphorylated ERK1/2 (p42/44) serves as loading control. Experiments with a different cell line (HepG) gave similar results.

**Figure 8:**
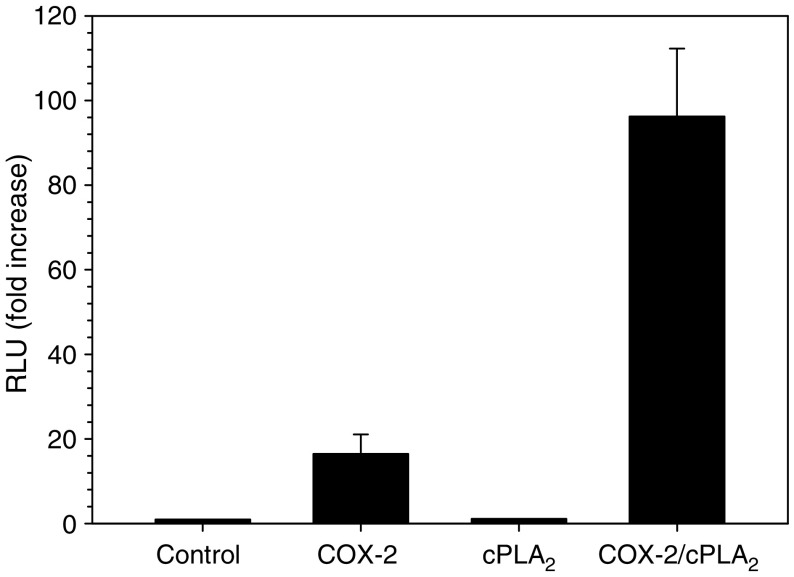
PPAR reporter assay using a DR1 construct. The fold increase is calculated for control and COX-2, cPLA and combined transfectants. Results are the mean from triplicates and the experiment was repeated once.

One possibility to counteract the antiproliferative effects of TGF-*β* is either blocking the intracellular signal transduction or upregulating a proliferation signal. The former has been shown to be mediated by Smad 7, although this effect has mostly been proven by the inhibition of the reporter. As no interference with the response of the reporter to TGF-*β* could be shown after COX-2 co-transfection, this pathway seems to be not effective. So the overexpression of a proliferation signal could be an alternative explanation. To test this, we investigated the changes in the expression of common growth factors for epithelial cells (HGF, EGF, TGF-*α*) by RT–PCR after COX-2 transfection. Whereas EGF and HGF were not induced after COX-2 expression, TGF-*α* could be readily detected by PCR after COX-2 transfection (Figure 5). In addition, the transfectants secreted considerable amounts of TGF-*α* into the supernatant (Figure 6). As a consequence, the cells also had a higher spontaneous phosphorylation of the p44/42 MAPK, which is a common pathway in growth factor stimulation (Figure 7, lane 1). To get an idea how the COX-2 signal might be transmitted, we measured PGE_2_ concentrations and also tested in a screen a pathway for lipid signalling. The prostaglandin concentrations in the medium were in the Mv1Lu cells 64.9 pg ml^−1^ in controls and 157.7 pg ml^−1^ in the COX-2 transfected cells, whereas in the human CRC-line HCT-15, the mean concentration was 100 pg ml^−1^ in controls and 450 pg ml^−1^ in the COX-2-expressing cells. This concentration was just below concentrations used to stimulate cells directly to proliferate. In addition, mRNA for the PGE receptors 2 and 4 was below the detection limit of the RT–PCR. Thus, it seems more likely that the signal might be transferred by an intracellular pathway. Indications for this could be seen in a screening for the reporter assay for lipid mediators. Transfecting a reporter with binding sites for PPAR/RXR together with COX-2 resulted in an increase in the luciferase activity compared with controls (Figure 8). Thus, it seems likely that an endogenous product generated or affected by the COX-2 overexpression fuelled the reporter expression.

## DISCUSSION

Overexpression of COX-2 has been associated with the risk for the development of many different tumours. As one possible outcome of the COX-2 overexpression, an escape of epithelial cells from the regulatory influence of TGF-*β* in the intestine has been discussed. This has been elegantly shown by the overexpression of COX-2 in a rat intestinal epithelial cell line, which resulted in a downregulation of TGF-*β*-receptor as well as an upregulation of bcl-2, which may stop growth inhibition by TGF-*β* as well as lower apoptosis and finally end in a higher proliferation of epithelial cells (Tsujii and DuBois, 1995). In contrast to these results, a loss of TGF-*β*-receptor expression could not be found after COX-2 overexpression in the experiments presented here. TGF-*β*, however, was no longer able to inhibit cell proliferation in Mv1Lu cells, a common indicator cell line for TGF-*β*-effects. Therefore, a mechanism different from the loss of receptor expression must be effective. The translation of the TGF-*β* signal into the cell involves several positive and negative regulators in the cytosol. The phosphorylation of Smad proteins and their translocation into the nucleus are indicative for an effective transfer of the signal. This pathway, however, was unaffected in Mv1Lu cells overexpressing COX-2. So how was the escape from inhibition mediated? Activating of inhibitors of the Smad pathway and Smad or even TGF-*β*-independent mechanisms might be responsible for the proliferation of the cells in the presence of TGF-*β* after COX-2 overexpression.

Direct inhibitory molecules, which interfere with the TGF-signalling pathway, have been described including Sno or Ski or more recently Akt (Conery *et al*, 2004; Remy *et al*, 2004). These molecules, however, act only in concert with Smad, for example, are bound to Smad and inhibit Smad translocation, which seems not to be affected in the experiments presented here. After TGF-*β* stimulation, the signal can be blocked by inhibitory factors like Smad 7 (Hayashi *et al*, 1997). The inhibitory effect of this pathway has been similarly proven by a reduction of the signal response of a reporter construct. As in the experiments presented here, COX-2 transfectants still respond to TGF-*β* with an increase of the reporter signal, direct inhibition of the Smad pathway seems to be unlikely.

The intracellular signalling of TGF-*β* consists of two well-defined pathways: one that includes the Smad signalling as outlined above (Massague *et al*, 2000) and a Smad-independent pathway (Derynck and Zhang, 2003). As the experiments show all activation steps in the Smad pathway even in the cells overexpressing COX-2, a Smad-independent pathway seems to be responsible for the escape of the cells from TGF-*β*-induced growth inhibition. There have been reports of crosstalk between the TGF-*β* signalling and an activation of receptor tyrosin kinases. The latter transmit quite often the signal of growth hormones and induce cell proliferation (Grunert *et al*, 2003) even in the presence of an antiproliferative milieu. In this regard, it has been postulated that the overexpression of an oncogene may override the TGF-*β* effects (Siegel and Massague, 2003). From this, it is an interesting finding that the COX-2-transfected cells display higher TGF-*α* expression/secretion and a higher activation of the ERK-pathway. The activation of the ERK pathway could be explained by the transactivation of the EGF receptor as has been shown for the stimulation with PGE_2_ (Pai *et al*, 2002). EGF-receptor activation leads to a signalling by the MAPK, which has been suggested to antagonise the TGF-*β* pathway (Lo *et al*, 2001) and induce the oncogene myc-expression (Alarcon-Vargas and Ronai, 2004), which may bypass the growth inhibition. In human cholangiocarcinoma cells, growth and invasive properties could be stimulated by PGE_2_/EP1 receptor mediated EGF-receptor activation (Han and Wu, 2005). However, although PGE could be found to be much higher in COX-2-transfected cells, it never reached the concentration used to induce transactivation. So a direct effect of the overexpressed TGF-*α* on the EGF-receptor seems more likely. An interrelation between COX-2 and growth factors has been shown before for TGF-*α* and amphiregulin (Coffey *et al*, 1997; Shao *et al*, 2004). In this regard, it is interesting that TGF-*α*-stimulated cells have been used for the isolation and cloning of COX-2 (DuBois *et al*, 1994) and shows the close interrelation between both systems. One must make the point, however, that the polarised secretion of the TGF-*α* together with the localised expression of the EGF-receptor may impede the growth stimulation in polarised epithelial cells. This may be changed dramatically in hyperproliferating cells as in adenomas or less differentiated cells, which may lose polarity (Huang *et al*, 1995). On the other hand, TGF-*β* has been shown to induce TGF-*α* expression with the consequence of EGF-receptor activation (Vinals and Pouyssegur, 2001) and an increase in c-myc oncogene expression (Skouteris and McMenamin, 1992), as we could demonstrate in the transfected cells here (Supplementary Figure 4). Although direct prostaglandin effects via exogenous receptor activation are a good explanation, endogenously produced lipid mediators may also mediate effects. In this regard, non-enzymatic products of the prostaglandin pathway like PGJ as well as arachidonic acid have been shown to trigger important signalling via PPAR activation (Dobbie *et al*, 2002; Laurora *et al*, 2003). The stimulation of a PPAR-responsive element in the DR-reporter after COX-2 overexpression demonstrates this pathway. There are reports of inhibitory effects of PPAR-*γ* on the TGF/Smad pathway and also on the block of TGF-*β*-induced mitoinhibition (Han *et al*, 2004; Zhao *et al*, 2006). Therefore, the direct PGE as well as the PPAR-mediated effects can explain the effects of COX-2 overexpression on the TGF-*β* pathway. This could, in addition to the reported loss of TGF-*β* receptor, explain how epithelial cells can escape from the growth regulation in the intestine ultimately leading to hyperproliferation and the formation of adenomas, which are prerequisite for carcinoma formation. Therefore, not only the inhibition of the cyclooxygenase is a good strategy for treating patients at risk but also a combination with PPAR inhibitor may boost the therapeutic repertoire.
