# Supplementary figures and images for: Cyclooxygenase-2 overexpression abrogates the antiproliferative effects of TGF-β
Source: Br J Cancer. 2007 Oct 23;97(10):1388–92. doi: 10.1038/sj.bjc.6604048 (PMC2360247; doi:10.1038/sj.bjc.6604048)

Supp Figure 1


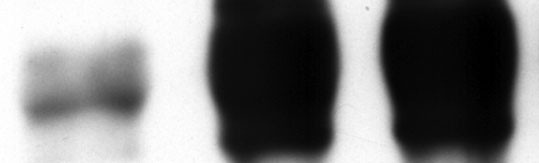


A.


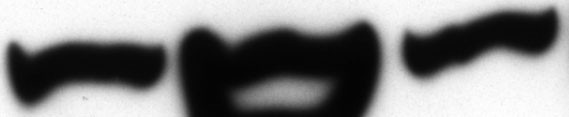


B.

1 2 3

Supplement: Supplementary Figure 1 [file 6604048x1.doc]

Supp. Figure 2

A.


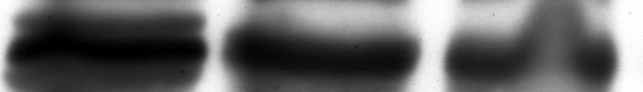


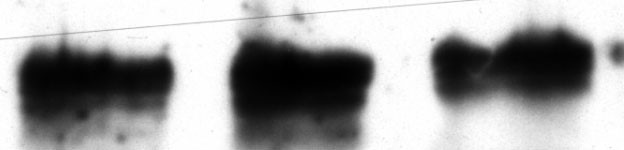


B.

1 2 3

Supplement: Supplementary Figure 2 [file 6604048x2.doc]

Supp Figure 4

1 2


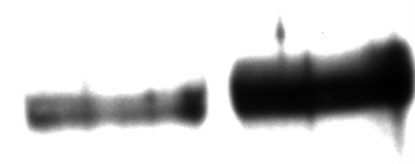

Supplement: Supplementary Figure 4 [file 6604048x4.doc]
