# Supplementary material for: Cyclooxygenase-2 overexpression abrogates the antiproliferative effects of TGF-β
Source: Br J Cancer. 2007 Oct 23;97(10):1388–92. doi: 10.1038/sj.bjc.6604048 (PMC2360247; doi:10.1038/sj.bjc.6604048)
Supplement: Supplementary Figure 3 [file 6604048x3.doc]

Supp. Figure 3

A B
